# The Assessment of the Dissociation of Intimacy: Development and Psychometric Properties of the Dissociation of Intimacy Questionnaire (DIQ)

**DOI:** 10.3390/ejihpe15120249

**Published:** 2025-12-06

**Authors:** Vincenzo Caretti, Eleonora Topino, Andrea Fontana, Adriano Schimmenti, Alessio Gori

**Affiliations:** 1 Department of Human Sciences, LUMSA University, Via della Traspontina, 21, 00193 Rome, Italya.fontana2@lumsa.it (A.F.); 2 Department of Human and Social Sciences, Mercatorum University, Piazza Mattei, 10, 00186 Rome, Italy; eleonora.topino@unimercatorum.it; 3 Department of Human and Social Sciences, UKE—Kore University of Enna, Cittadella Universitaria, 94100 Enna, Italy; 4 Department of Health Sciences, University of Florence, Via di San Salvi 12, Pad. 26, 50135 Firenze, Italy; 5 Integrated Psychodynamic Psychotherapy Institute (IPPI), Via Ricasoli 32, 50122 Florence, Italy

**Keywords:** alexithymia, attachment, closeness, dissociation, interpersonal functioning, intimacy

## Abstract

Intimacy is a core domain of personality functioning, but it can be compromised by defensive and dissociative processes. Given this, the present study aimed to develop and validate the Dissociation of Intimacy Questionnaire (DIQ), a multidimensional self-report instrument assessing dissociative disruptions of intimacy. The DIQ consists of two parallel forms (“Me with Others” and “Others with Me”) developed to capture five distinct dimensions of the dissociation of intimacy: emotional, psychological, physical, social, and sexual. The sample comprised 500 participants (74% females; *M_age_* = 31.92 years, *SD* = 12.78) recruited through online snowball sampling. Exploratory and confirmatory factor analyses were conducted, alongside reliability and validity testing. Both exploratory and confirmatory analyses supported the five-factor structure of the DIQ, with satisfactory model fit indices. Internal consistency was good across all subscales. Convergent validity was demonstrated through significant positive correlations with insecure attachment, alexithymia, somatoform dissociation, and impairments in personality functioning. The DIQ is a robust and clinically relevant tool for assessing dissociation of intimacy. Its multidimensional and mirror structure allows for a detailed understanding of impairments in attitudes to closeness and provides a valuable framework for both research and psychotherapy.

## 1. Introduction

### 1.1. The Construct of Dissociation of Intimacy

Dissociation of intimacy (DOI) is a clinical and theoretical construct denoting a defensive and dysfunctional mode of relating in close relationships, characterized by a disconnection (conscious or unconscious) between the self and the relational experience across emotional, psychological, social, physical, and sexual domains. This transdiagnostic pattern is frequently observed in individuals with histories of traumatic attachment and emotion dysregulation (Schore, 2003). In line with contemporary clinical and empirical literature, dissociation more broadly can be understood as a psychic process that is activated in response to severe psychological trauma and overwhelming affect, through which behaviors, feelings, memories, and bodily states become reciprocally disconnected in order to protect the integrity of the self (Bowlby, 1969; Putnam, 1992; Schimmenti & Caretti, 2016; Schimmenti, 2017). From an attachment-based and developmental perspective, dissociation represents a paradoxical adaptation to caregiving environments that are simultaneously needed and feared: by excluding from consciousness those experiences that are incompatible with the ongoing relationship, the child maintains a sense of continuity at the price of “amputating” aspects of subjective experience. Building on this framework, we define DOI more specifically as a defensive and dysfunctional mode of relating in close relationships, whereby the individual selectively severs emotional, bodily, and cognitive engagement in contexts that would normally support mutual vulnerability, co-regulation, and shared meaning-making. Intimacy is one of the relational spaces most threatened by developmental trauma, because proximity is implicitly anticipated as a source of injury, rejection, or abandonment rather than safety (Schimmenti, 2012, 2013). Clinically, this can manifest as a structural split between different tracks of relating: on the one hand, relationships perceived as safe, caretaking, and reliable but emotionally flattened; on the other hand, sexual scenarios, behaviors, or fantasies that are intense yet not mentalized, depersonalized, or experienced “as if from the outside,” and not integrated with genuine closeness or mutual recognition. Patients often describe experiences such as “it is as if it wasn’t me”, “as if my body went on by itself” or “I feel present only with my mind, not with my body”. DOI thus refers to a trauma-based disruption of the capacity to inhabit intimate relationships with an integrated sense of self and other across emotional, psychological, physical, social, and sexual domains.

The antecedents of DOI can be traced back to traumatic attachment experiences in which caregivers are chronically misattuned, rejecting, or frightening, and in which attempts at dyadic repair repeatedly fail (Schore, 2003, 2012; Tronick, 1989). Under these conditions, the child’s emerging representations of self–other relatedness become organized around expectations of danger and non-repair, fostering chronic mistrust and shame in the interpersonal domain. In adulthood, these internal scripts are often reactivated when intimacy implies dependence, vulnerability, or embodied closeness. The resulting outcomes include the construction of relational barriers that prevent genuine proximity, controlling or punitive strategies that undermine reciprocity, and impulsive or egocentric behaviors that prioritize self-protection over mutual attunement. Over time, such patterns contribute to social withdrawal, pseudointimate bonds, and broader impairments in personality functioning, as reflected in the AMPD domain of intimacy (APA, 2013, 2022). In this perspective, DOI encompasses both (a) defensive relational patterns such as avoidance, mistrust, and difficulty in emotional disclosure, and (b) more overt dissociative experiences of not being fully present, feeling detached, or experiencing intimacy as unreal.

### 1.2. Dissociation of Intimacy and Related Constructs

While DOI is conceptually related to established constructs such as insecure attachment, fear of intimacy, and general dissociation, it is not reducible to any of them. Although partially overlapping, fear of intimacy scales typically taps conscious worries and anticipatory anxiety about getting close to others or being known in depth, and the tendency to avoid self-disclosure to prevent rejection or loss of control (Senese et al., 2020). By contrast, DOI focuses on experiential discontinuities that arise when intimacy actually occurs, even in individuals who consciously desire closeness or value romantic bonds. Whereas fear of intimacy primarily indexes what people expect or fear *before* entering intimacy, DOI addresses what happens to the sense of self and to affective engagement *within* intimate encounters, including sudden numbing, depersonalization-like experiences, or a subjective sense of “going away” in the presence of the other.

Similarly, DOI is conceptually related but not equivalent to adult attachment anxiety and avoidance. Attachment avoidance reflects a generalized deactivating strategy characterized by discomfort with dependence, emotional distance, and a preference for autonomy, while attachment anxiety involves hyperactivation of the attachment system and chronic concern about abandonment. Instruments such as the Experiences in Close Relationship (ECR; Fraley & Shaver, 2000) capture these dimensions as relatively stable relational orientations. In contrast, DOI targets micro-temporal and dissociative shifts in agency, ownership, and affective presence that occur when the attachment system is activated. An individual may score as avoidant and maintain a coherent, non-dissociative sense of self; another may show moderate avoidance but, once intimacy is established, repeatedly experience episodes of emotional shutdown or feeling “not fully there” with the partner. Thus, DOI cuts across attachment styles and helps to specify *how* attachment dynamics are enacted at the level of lived self-experience in close relationships, rather than merely *whether* one is generally anxious or avoidant about mental states connected to attachment.

With respect to general dissociation, classical measures such as the Dissociative Experiences Scale (DES-II; Saggino et al., 2020) assess a broad spectrum of phenomena—absorption, depersonalization/derealization, and amnesia—across multiple domains of identity, memory, perception and consciousness. These indices are intentionally content-general, aggregating dissociation that may occur in everyday life, under stress, in solitude, or in a wide variety of interpersonal and non-interpersonal contexts. DOI, by contrast, is functionally more circumscribed: it refers to dissociative experiences that are specifically triggered by intimacy and attachment-relevant proximity, and that involve a disconnection between implicit bodily/sensory markers of safety, pleasure and trust and the presence of a significant other. While it may share phenomenological features with depersonalization or derealization, it is defined by its relational trigger and interpersonal meaning—namely, the deactivation or “sabotage” of mature dependency and authentic closeness.

Finally, DOI is related to, but distinct from, impairments in intimacy as described in the AMPD Criterion A (APA, 2013). AMPD intimacy primarily captures structural and trait-like capacities for forming and maintaining close relationships, such as mutuality, depth, and durability of bonds. It tells us whether individuals are generally capable of engaging in reciprocal, caring, and sustained intimacy. DOI, instead, is a process-oriented and situational construct that describes how intimacy is experienced and regulated in vivo: whether closeness is associated with stable engagement or with recurrent episodes of emotional disconnection, self-alienation, and relational shutdown. Two patients may display similar AMPD intimacy impairments in terms of chronic difficulties in forming satisfying relationships, yet differ markedly in the presence or absence of acute dissociative states in intimacy; conversely, DOI may be observable also in individuals who do not reach the threshold for personality disorder but show trauma-related or defensive disconnections in close bonds.

From a broader perspective on the sense of self, recent work has proposed that self-experience can be understood as the dynamic integration of at least two core components (Di Plinio et al., 2024): identity (the feeling of being a temporally continuous, self-referential subject) and agency (the feeling of being the author of one’s actions, thoughts, and their consequences). This perspective emphasizes a pre-reflective, embodied level of selfhood that is continuously embedded in the world through perception and action. Di Plinio et al. (2024) have recently operationalized this view by outlining a unidimensional trait-self dimension combining identity and agency, which showed meaningful associations with psychosis-like experiences, schizotypal traits, and hopelessness, supporting the idea that alterations in basic self-experience are relevant for mental health. Within this broader scaffold, DOI can be conceptualized as a *domain-specific disturbance of self-experience* that emerges when different motivational systems (e.g., attachment- and sexuality-related systems) are activated in intimate relationships. Rather than indicating a global erosion of minimal selfhood, DOI describes difficulties in maintaining an *integrated sense of being oneself* (identity) and *acting from oneself* (agency) precisely in contexts of emotional bonding. In phenomenological terms, this corresponds to a relationally cued self-fragmentation, in which the coherence between identity and agency is selectively compromised in intimate contexts, likely reflecting the long-term impact of traumatic attachment experiences on the organization of self and relationships. In this vein, DOI reflects situational failures in the experience of “mineness” and control specifically in the context of intimate interactions. In those moments, the individual may continue to behave in a socially appropriate way, yet subjectively feels less present, less agentic, and less connected both to self and to the other. In this sense, DOI bridges trauma- and attachment-informed models of dissociation with contemporary views on the dynamic, context-sensitive sense of self, adding a relationally focused and clinically tractable construct to the assessment of self–other functioning in personality pathology.

### 1.3. Theoretical Foundations of Dissociation of Intimacy

The clinical picture of DOI can be illuminated by converging psychodynamic, attachment, and neurobiological models. Traumatic attachment involves developmental experiences where the caregiving figure, meant to foster safety, also acts as a source of fear, shame, or confusion (Farina & Schimmenti, 2025). This paradox generates deep ambivalence and undermines the ability to experience closeness as safe. In adult relationships, these unresolved relational templates often re-emerge as dissociative strategies—emotional numbing, mistrust, withdrawal—that protect the self from anticipated injury (Caretti et al., 2018; Schimmenti, 2023).

The antilibidinal ego theorized by Fairbairn (1944/1952) provides an intrapsychic framework for understanding how intimacy becomes dissociated. In Fairbairn’s view, a persecutory internal object, formed through the internalization of rejecting or punishing caregivers, organizes a defensive structure (the Internal Saboteur) that inhibits libidinal desire and trust in closeness. Intimacy thus becomes a scene in which longing for connection is immediately countered by internal attack and withdrawal, setting the stage for dissociative shutdown.

Bowlby’s (1973) attachment theory offers further insight. When proximity to caregivers repeatedly evokes fear, abandonment, or unpredictability, intimacy may come to be experienced as dangerous, precipitating unconscious relational strategies (avoidance, withdrawal, controlling behavior) that defend against vulnerability and loss (Reisz et al., 2018). Accordingly, “fear of intimacy” represents a key feature of insecure attachment styles (Bifulco et al., 2008). DOI can be seen as a more radical outcome along this trajectory, in which the attachment system is not only hyper- or de-activated, but partially disconnected from embodied emotional experience.

Kernberg (1975), Kernberg and Caligor (2005) placed the failure of relational integration at the center of personality pathology. The inability to synthesize idealized and persecutory representations of self and others leads to identity diffusion and primitive defenses, especially splitting and dissociation. These impairments compromise the formation of mature, reciprocal intimacy, fostering unstable bonds marked by mistrust, fear, and affective dysregulation—conditions under which dissociative responses in intimacy are particularly likely.

Furthermore, Bromberg (1998, 2006) contributed a relational–dissociative model of the mind, in which dissociation is a ubiquitous organizing principle rather than a rare disruption. For Bromberg, the self is composed of multiple self-states that alternate depending on the relational field and emotional safety. When intimacy evokes threat, shame, or a revival of traumatic intersubjective experiences, certain self-states become sequestered or disowned. From this vantage point, DOI reflects the non-integrated multiplicity of the self in relation: a person may long for closeness yet flee from it behaviorally, without conscious awareness of the contradiction. The therapeutic process involves helping the patient stand “in the spaces” between these dissociated self-states, tolerating relational presence without collapsing into avoidance or fragmentation.

These psychodynamic accounts converge with Stephen Porges’ Polyvagal Theory (Porges, 2011), which provides a neurophysiological framework for understanding how the autonomic nervous system supports or disrupts relational functioning. The autonomic nervous system is hierarchically organized into three response systems: a ventral vagal system associated with safety, social engagement, and co-regulation; a sympathetic system mobilized in fight-or-flight responses; and a dorsal vagal system mediating immobilization, shutdown, and dissociative collapse. At the core of this theory is *neuroception*, the nervous system’s implicit and automatic detection of safety or threat without conscious appraisal (Porges, 2011, 2025). When neuroception registers safety, the ventral vagal system supports bodily presence and intimacy; when it detects threat, the social engagement system is inhibited, and older defense circuits dominate. Within this polyvagal framework, DOI can be understood as a neurobiologically mediated strategy of protection: proximity, touch, or emotional availability activate implicit memories of relational threat, and the autonomic nervous system inhibits connection through partial or full disengagement. These responses often occur beneath awareness, manifesting in the therapy room as sudden inaccessibility, dissociative silence, withdrawal of gaze, flattened affect, or the subjective sense of being “*not fully there*.” The failure to activate the ventral vagal system, due to early attachment trauma or chronic stress, precludes the development of co-regulation, which is essential for enduring intimacy; the individual may appear avoidant or aloof, when in fact they are neurobiologically unable to tolerate proximity without triggering physiological threat responses (Dana, 2018; Porges, 2021).

Integrating Porges’ physiological insights, Schore (2003, 2012) has shown how early relational trauma and caregiver misattunement shape the development of right-brain regulatory systems. The orbitofrontal cortex and right-lateralized limbic structures, essential for affect regulation and relational engagement, are sculpted by emotionally attuned interactions in infancy. When these interactions are consistently neglecting or abusive, the developing brain organizes itself defensively, privileging dissociation, hyperarousal, or shutdown as adaptive mechanisms; DOI can thus be seen as a neurobiological and relational adaptation to early environments in which proximity was unpredictably paired with danger.

### 1.4. Assessing Dissociation of Intimacy

The DSM-5 Alternative Model for Personality Disorders (AMPD) (APA, 2013) offers a dimensional framework that reconceptualizes personality pathology not merely as a categorical syndrome, but as a spectrum of impairment in core psychological functions. The AMPD defines Level of Personality Functioning (Criterion A) as the central organizing construct, composed of two domains: Self Functioning (Identity and Self-Direction) and Interpersonal Functioning (Empathy and Intimacy). In particular, intimacy is operationalized as the capacity to form and maintain close, stable, and mutually satisfying relationships characterized by depth of connection, mutual regard, and emotional sharing. Impairments in intimacy may manifest as detachment, superficiality, avoidance of closeness, or chronic mistrust in relationships. According to the AMPD, disturbances in intimacy reflect foundational disruptions in personality integration, and are therefore considered central diagnostic indicators of personality dysfunction. The SCID-5-AMPD (APA, 2013, 2022; Bender et al., 2018), a semi-structured clinical interview developed for assessing the AMPD model, includes a focused evaluation of the intimacy domain. This assessment explores the individual’s ability to establish meaningful emotional bonds, tolerate relational vulnerability, and sustain reciprocity.

DOI, though not explicitly labeled in the AMPD, aligns closely with impairments in this domain, particularly when social situations evoke relational avoidance, emotional disconnection, or pseudointimacy. From this vantage point, the Dissociation of Intimacy Questionnaire (DIQ) responds to a significant gap in available instruments by offering a theory-driven, empirically grounded, and clinically sensitive measure of the dissociative phenomena that compromise intimacy. The DIQ captures a multidimensional pattern including difficulties in emotional disclosure and trust, avoidance of romantic and physical closeness, interpersonal detachment, and dissociative experiences of “not being fully present”, bodily numbing, or feeling like an external spectator in intimate and sexual situations, assessed both from the perspective of “myself with others” and “others with me”. Unlike general interpersonal functioning scales, the DIQ focuses specifically on how proximity, vulnerability, and affective closeness may trigger defensive dissociation, and how these responses are embodied, relationally enacted, and interpersonally perceived.

### 1.5. The Present Research

From a clinical standpoint, assessing dissociation of intimacy is essential for recognizing the defensive, neurobiological, and representational dynamics that interfere with closeness. Whether via internal persecutory structures, attachment-based fear, or dissociated self-states, these mechanisms often remain invisible in clinical formulations unless explicitly addressed. Despite the growing theoretical interest in trauma-related dissociation, attachment disturbances, and impairments in the intimacy domain of personality functioning, there is currently no instrument specifically designed to assess dissociative disruptions of intimacy across emotional, psychological, physical, social, and sexual dimensions. Existing measures capture either conscious fears and avoidance of closeness, general dissociative symptomatology, or broad trait-level capacities for intimacy, but they do not directly tap the relationally triggered, state-like breakdowns in self–other experience that characterize dissociation of intimacy. On this basis, the present study aimed to validate the factorial structure, internal consistency, and psychometric properties of the Dissociation of Intimacy Questionnaire (DIQ) in a large non-clinical sample. The instrument seeks to operationalize dissociation of intimacy and contribute to the advancement of trauma-informed, relational, and dimensional approaches to personality assessment.

## 2. Materials and Methods

### 2.1. Participants

A total of 500 subjects were involved in the study (74% female; 26% male). Participants’ ages ranged from 18 to 78 years (*M* = 31.92, *SD* = 12.78). Regarding marital status, most participants were single (69.0%), followed by cohabiting (12.4%) and married (14.0%), while a minority reported being divorced (2.8%), separated (1.6%), or widowed (0.2%). Concerning educational level, 34.8% of respondents had a high school diploma. With respect to employment status, 32.8% of participants were students, 23.0% employees, and 19.8% student-workers (see Table 1).

### 2.2. Procedure and Ethics

Participants were recruited online through a snowball sampling procedure and completed an anonymous self-report survey on the Google Forms platform. Eligibility criteria required participants to be at least 18 years old, to have sufficient proficiency in Italian, and to provide informed consent. These inclusion criteria were implemented directly in the survey so that individuals who did not meet them could not proceed with the questionnaires. Because of the snowball recruitment strategy, it was not possible to determine the total number of individuals initially reached or to compute a response rate. No additional exclusion criteria or data-trimming procedures were applied. The questionnaire included demographic information (sex, age, marital status, occupation, and educational level) along with the study measures. Informed consent was obtained electronically prior to participation. The study was approved by the Institutional Ethical Committee of one of the authors’ affiliated institutions.

### 2.3. Development of the Dissociation of Intimacy Questionnaire (DIQ)

The *Dissociation of Intimacy Questionnaire (DIQ)* was elaborated with the specific aim of operationalizing the construct of dissociation of intimacy within a clinically sensitive and empirically grounded framework. The development of the DIQ was guided by three primary objectives: (a) *Conceptual accuracy*, i.e., providing a measure closely aligned with psychodynamic, attachment, and neurobiological models of dissociation, thus reflecting the complexity of defensive strategies observed in clinical practice; (b) *Bidirectional assessment*, through the creation of a *mirroring structure* with two complementary versions (*Me with others*/*Others with me*), in order to capture both self-perceptions and the individual’s representation of others’ relational stance; (c) *Clinical utility*, ensuring that the instrument could be used both in research and in therapeutic contexts to explore dissociative dynamics in intimacy, thereby filling a gap left by broader measures of interpersonal functioning, such as those included in the DSM-5 Alternative Model for Personality Disorders (APA, 2013, 2022; Bender et al., 2018).

The DIQ is articulated into five factors, each designed to capture a distinct domain of relational dissociation:

1. *Barriers to Closeness* corresponds to difficulties in expressing and sharing affective states with others, stemming from fear of judgment, rejection, or emotional invalidation. Whereas emotional intimacy normally entails safety in disclosing personal feelings (Gaia, 2002; Weber et al., 2004), its dissociative form is characterized by avoidance of self-disclosure and affective withdrawal.
2. *Relational Mistrust* reflects impairments in reciprocal trust, empathy, and mental attunement. While psychological intimacy supports mental closeness and repair of ruptures in interpersonal engagement (Collins & Feeney, 2004; Rosbrow, 2014), its dissociation involves mistrust, suspicion, and fear of abandonment, undermining the possibility of secure relational bonds.
3. *Physical Detachment* denotes difficulties in tolerating bodily proximity, touch, and affectionate gestures. Physical intimacy typically implies tenderness, closeness, and bodily presence (Maclaren, 2014), but its dissociation manifests as rigidity, emotional detachment, or the subjective sense of not being fully present during physical contact.
4. *Social Misattunement* concerns detachment in group and collective interactions. Social intimacy refers to shared interests, hobbies, or projects that sustain collaboration and belonging (Baumeister & Leary, 2017; Myers, 2003); in its dissociative form, however, the individual feels estranged, bored, or peripheral, as if observing rather than participating in the social exchange.
5. *Sexual Disembodiment* captures dissociative experiences specific to the sexual domain. Sexual intimacy normally involves desire, erotic involvement, and the integration of bodily and affective presence (Ferreira et al., 2012). In its dissociative form, it is marked by a lack of bodily awareness, the perception of the sexual experience as unreal, or the feeling of being an external observer during the encounter.

The development of the DIQ items was carried out through a multi-phase process, in line with international guidelines for scale construction (Netemeyer et al., 2003). After a comprehensive review of the literature on the theme, a preliminary pool of items was generated by four of the coauthors (VC, AF, AS, and AG) to reflect the five domains of intimacy (emotional, psychological, physical, social, and sexual) in their dissociative manifestations. To ensure content validity, a series of focus groups was organized with a panel of experts in clinical psychology and psychotherapy. Each item was carefully discussed with respect to its clarity, theoretical relevance, and clinical usefulness. A distinctive feature of the DIQ is its mirroring structure, which was established from the very beginning of item development. Specifically, for each of the five factors, parallel items were constructed to capture both perspectives: (a) Me with others (self-perceptions of one’s behavior in intimate contexts) and (b) Others with me (perceptions of others’ attitudes toward oneself). All items were written in simple, jargon-free language to ensure accessibility. Responses are collected using a 5-point Likert scale (1 = Not at all; 2 = A little; 3 = Somewhat; 4 = Rather much; 5 = Very much), a format chosen for its clarity, ease of interpretation, and widespread use in psychometric assessment. Higher scores reflect greater dissociation in the corresponding domain of intimacy.

### 2.4. Measures

The survey included sociodemographic questions, the DIQ, and several validated self-report instruments administered as external criteria to evaluate the construct validity of dissociation of intimacy. These measures were selected because they assess constructs that are theoretically proximal to intimacy disruptions (i.e., adult attachment patterns, difficulties in identifying and describing emotions, and impairments in personality functioning) and because they are widely used and well-validated in both their original and Italian versions.

The *Dissociation of Intimacy Questionnaire (DIQ)* is a 30-item self-report questionnaire designed to assess dissociation of intimacy. It is composed of two mirroring versions: (a) Me with others, which evaluates how individuals perceive their own behaviors and experiences in contexts of intimacy, and (b) Others with me, which assesses how individuals perceive the attitudes and behaviors of others toward them in similar contexts. Each version comprises 15 items, distributed equally across five factors, which were labelled as *Barriers to Closeness, Relational Mistrust*, *Physical Detachment*, *Social Misattunement*, and *Sexual Disembodiment*. Items are rated on a 5-point Likert scale (1 = Not at all, 2 = A little, 3 = Somewhat, 4 = Rather much, 5 = Very much). For each factor, a subscale score is computed by summing the three corresponding items, with higher scores indicating greater dissociation in that domain. In addition, total scores can be calculated separately for each version (Me with others and Others with me), as well as a global score reflecting overall dissociation of intimacy.

The *Relationship Questionnaire* (*RQ*; Bartholomew & Horowitz, 1991; Carli, 1995) is a 4-item self-report instrument developed to assess adult attachment styles. Each item represents one of four prototypical patterns: secure, preoccupied, dismissing, and fearful attachment. Participants rate the degree to which each description corresponds to their typical relational style on a 7-point Likert scale, ranging from 1 (*It does not describe me at all*) to 7 (*It very much describes me*). Responses can be used to derive continuous scores along two higher-order attachment dimensions: attachment anxiety and attachment avoidance. Given the scoring procedure, which combines single-item ratings through a predefined algorithm, internal consistency indices (e.g., Cronbach’s alpha) cannot be computed.

The *Somatoform Dissociation Questionnaire*—*short version* (*SDQ—5*; Nijenhuis et al., 1997; Schimmenti & Mulé, 2007) is a 5-item screening instrument for dissociative disorders. Items are rated on a 5-point Likert-type scale, ranging from 1 (*This applies to me not at all*) to 5 (*This applies to me extremely*), yielding total scores between 5 and 25. In the present study, the SDQ-5 showed acceptable internal consistency (*α* = 0.692).

The *Twenty-Item Toronto Alexithymia Scale* (*TAS-20*; Bagby et al., 1994a, 1994b; Bressi et al., 1996) is a 20-item self-report scale designed to assess alexithymia. Items are rated on a 5-point Likert scale, from 1 (*Strongly disagree*) to 5 (*Strongly agree*). The TAS-20 provides a total score as well as three subscales: difficulty identifying feelings (DIF), difficulty describing feelings (DDF), and externally oriented thinking (EOT). In the present study, the Italian version was used, and internal consistency was satisfactory for the total score (*α* = 0.838), DIF (*α* = 0.868), and DDF (*α* = 0.780), while lower for EOT (*α* = 0.583).

The *Level of Personality Functioning Scale* (*LPFS-BF*; Weekers et al., 2019; Gritti et al., 2025) is a 12-item self-report instrument assessing the severity of personality dysfunction as described in Section III if the DSM-5 Alternative Model for Personality Disorders (APA, 2013, 2022). The measure yields two subscales, with higher scores indicating greater impairment in personality functioning: Self-functioning and Interpersonal functioning. Items are rated on a 4-point Likert scale, from 1 (*Completely untrue*) to 4 (*Completely true*). In the present study, the Italian version was used, and internal consistency was good (Self-functioning, *α* = 0.882; Interpersonal functioning, *α* = 0.748).

### 2.5. Data Analysis

Statistical analyses were performed with JASP (JASP Team, 2025). The survey platform was configured so that all items were mandatory, and the questionnaire could not be submitted unless all questions had been answered. As a result, the final dataset consisted of 500 complete protocols with no missing data on the study variables. All available cases were therefore retained for the subsequent analyses. Univariate normality of item scores was screened through skewness and kurtosis. Following widely used guidelines, values with absolute skewness < |3.0| and absolute kurtosis < |10.0| (R. B. Kline, 2015) were considered acceptable in medium-to-large samples. The Kaiser–Meyer–Olkin (KMO) measure and Bartlett’s test of sphericity were computed to evaluate the suitability of the correlation matrix for factor extraction; KMO values ≥ 0.70 together with a significant Bartlett’s test (*p* < 0.001) were taken as evidence of adequacy (Kaiser, 1974; Mulaik, 2009). To examine the latent structure of the DIQ, the total sample (*N* = 500) was randomly divided into two subsamples of equal size. In the first subsample (*n* = 250), Exploratory Factor Analyses (EFAs) were conducted on the correlation matrix, using Principal Axis Factoring as the extraction method and an oblique rotation (oblimin), given the expected correlations among factors. Consistent with the a-priori theoretical model, the number of factors was fixed to five. Factor loadings ≥ 0.30 were considered salient (P. Kline, 2014). In the second subsample (*n* = 250), Confirmatory factor analyses (CFAs) were conducted on polychoric correlation matrices using the robust weighted least squares estimator (WLSMV), which is recommended for five-point Likert-type ordinal indicators (Li, 2016). Model fit was evaluated using the following indices: the Comparative Fit Index (CFI), Tucker–Lewis Index (TLI), and Goodness-of-Fit Index (GFI), with values ≥ 0.90 indicating acceptable fit (Byrne, 2010; Hu & Bentler, 1999; R. B. Kline, 2015); the Standardized Root Mean Square Residual (SRMR) and Root Mean Square Error of Approximation (RMSEA) with values ≤ 0.08 denoting adequate fit (Hu & Bentler, 1999). Concerning RMSEA, the 90% confidence interval was also examined, with upper bounds of the interval up to 0.08 considered indicative of acceptable fit, whereas values up to 0.10 were interpreted as reflecting an approximate but still reasonable fit to the data (MacCallum et al., 1996). In addition, standardized residual covariances were inspected to detect potential localized areas of misfit, with absolute values greater than approximately 2.0 considered indicative of possible local misspecification (R. B. Kline, 2015). To further examine the robustness of the measurement model, multigroup confirmatory factor analyses (MGCFA) were conducted to test measurement invariance across gender. These analyses followed the hierarchical sequence of increasingly restrictive models typically recommended in the literature (Byrne, 2012; Meredith, 1993): configural invariance (same factorial structure across groups, no equality constraints), metric invariance (factor loadings constrained to equality), scalar invariance (both factor loadings and intercepts constrained), and structural invariance (equality of latent variances, covariances, and means across groups). Model comparisons were evaluated using changes in fit indices, as recommended in the international literature, rather than relying exclusively on the *χ*^2^ difference test, which is overly sensitive to sample size (Cheung & Rensvold, 2002; Chen, 2007). Specifically, invariance was considered tenable when the change in the Comparative Fit Index (ΔCFI) did not exceed 0.01, and when the change in the Standardized Root Mean Square Residual (ΔSRMR) was ≤ 0.03 for metric invariance and ≤ 0.01 for scalar invariance (Cheung & Rensvold, 2002; Chen, 2007). To assess the internal consistency of each DIQ part, Cronbach’s alpha (α; Cronbach, 1951) and McDonald’s omega (ω; McDonald, 2013) coefficients were calculated. Correlations between corresponding Part A (“Me with Others”) and Part B (“Others with Me”) subscales were computed to evaluate the convergence between the two mirrored perspectives. Following conventional benchmarks (Cohen, 1988), correlations around 0.10, 0.30, and 0.50 were interpreted as small, medium, and large, respectively. In the present context, medium-to-large positive correlations (approximately *r* = 0.30–0.70), clearly below unity and not approaching levels typically associated with redundancy (e.g., r ≥ 0.80; R. B. Kline, 2015) were considered evidence that the mirrored subscales capture related but non-identical dimensions. Discriminant validity among the factors was further assessed using the heterotrait–monotrait ratio of correlations (HTMT). Values below 0.90 were considered indicative of satisfactory discriminant validity (Henseler et al., 2015). Furthermore, Pearson’s correlations were used to examine convergent validity.

## 3. Results

The item analysis suggested an approximately normal distribution of responses. Skewness values ranged from −0.926 (DIQ_A5) to +2.356 (DIQ_B11), remaining below the conservative cut-off of 3. Absolute kurtosis values varied between 0.409 (DIQ_A3) and 6.661 (DIQ_B11), remaining below the recommended cut-off of |10.0|. These results supported the suitability of the data for factor analysis.

Kaiser–Meyer–Olkin (KMO) values were 0.894 for Part A and 0.887 for Part B, exceeding the recommended threshold of 0.70. In addition, Bartlett’s test of sphericity was statistically significant for both parts (Part A: *χ*^2^(105) = 3865.05, *p* < 0.001; Part B: *χ*^2^(105) = 3092.49, *p* < 0.001), further confirming the adequacy of the correlation matrices for factor analysis. Concerning EFAs (see Table 2), for Part A (Me with others), five interpretable factors emerged, explaining 62.0% of the total variance. For Part B (Others with me), five interpretable factors also emerged, explaining 55.1% of the total variance.

**Figure 1:**
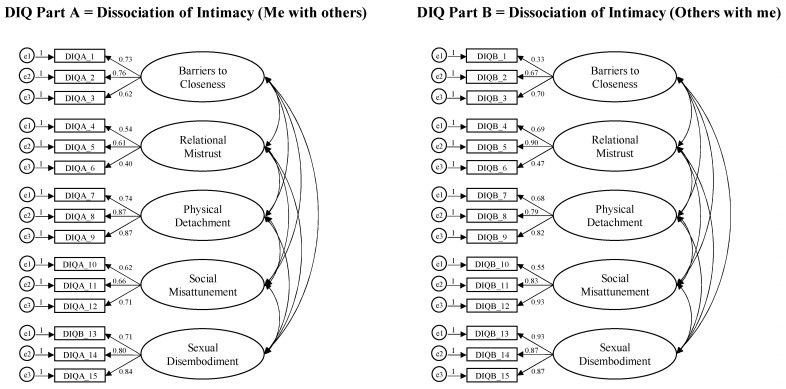
Graphical representation of factor structure for both DIQ Part A (Me with Others) and DIQ Part B (Others with me).

The CFAs confirmed the five-factor structure of each part of the DIQ (see Figure 1). For Part A, the chi-square was statistically significant, *χ*^2^(80) = 131.017, *p* < 0.001, with the following fit indices: CFI = 0.98, TLI = 0.97, GFI = 0.99, RMSEA = 0.05 (90% CI [0.034, 0.066]), and SRMR = 0.06. For Part B, the chi-square was also significant, *χ*^2^(80) = 173.610, *p* < 0.001, with fit indices showing satisfactory values: CFI = 0.95, TLI = 0.93, GFI = 0.99, RMSEA = 0.07 (90% CI [0.055, 0.083]), and SRMR = 0.06.

For Part A, standardized inter-factor covariances (i.e., latent correlations) ranged from 0.36 (between Barriers to Closeness and Relational Mistrust) to 0.76 (between Physical Detachment and Sexual Disembodiment). For Part B, standardized inter-factor covariances ranged from 0.65 (between Relational Mistrust and Social Misattunement) to 0.89 (between Social Misattunement and Sexual Disembodiment). For both Part A and Part B, all absolute residuals were below the conventional threshold of 2.00, indicating no evidence of localized model misfit for either form.

Multigroup CFA was conducted to test measurement invariance across gender (see Table 3). 

For Part A, the configural model showed acceptable fit indices (*χ*^2^(160) = 311.74, *p* < 0.001, CFI = 0.93, TLI = 0.90, GFI = 0.94, RMSEA = 0.09, SRMR = 0.06). Constraining factor loadings (metric invariance) led to a small decrease in fit (ΔCFI = −0.02; ΔSRMR = −0.02), while adding intercept constraints (scalar invariance) did not further reduce fit (ΔCFI = 0.00; ΔSRMR = −0.01). At the structural level (*χ*^2^(195) = 399.92, *p* < 0.001, CFI = 0.91, TLI = 0.91, GFI = 0.92, RMSEA = 0.08, SRMR = 0.11), a further decrease in fit was observed (ΔCFI = −0.01; ΔSRMR = +0.04), indicating only partial support for structural invariance in Part A. To clarify this result, item-level diagnostics were examined for the structural model. For women, the largest modification indices were concentrated on a small subset of items, particularly DIQ_A8, DIQ_A9, DIQ_A2, and DIQ_A3, which showed the highest indices for potential cross-loadings (e.g., DIQ_A8 and DIQ_A9 on the Barriers to Closeness factor, DIQ_A2 and DIQ_A3 on the Physical Detachment and Sexual Disembodiment factors, and DIQ_A4 and DIQ_A5 on the Social Misattunement factor). In addition, several residual covariances among items involving DIQ_A2, DIQ_A7, DIQ_A14, DIQ_A3, DIQ_A8, and DIQ_A15 (e.g., DIQ_A2–DIQ_A7, DIQ_A2–DIQ_A14, DIQ_A3–DIQ_A8, DIQ_A1–DIQ_A3, DIQ_A7–DIQ_A15) showed relatively high modification indices in the female group. For men, a similar but less pronounced pattern emerged. The largest cross-loading modification indices again involved a limited number of items, most notably DIQ_A1, DIQ_A3, DIQ_A8, DIQ_A9, DIQ_A6, and DIQ_A13 (e.g., DIQ_A1 and DIQ_A3 on the Social Misattunement factor, DIQ_A8 and DIQ_A9 on the Barriers to Closeness factor, DIQ_A6 on the Social Misattunement factor, DIQ_A13 on the Sexual Disembodiment factor). The highest residual covariances were observed for pairs such as DIQ_A13–DIQ_A15, DIQ_A1–DIQ_A6, and DIQ_A6–DIQ_A10. Overall, these local departures from invariance are limited in number and magnitude and do not form a pervasive pattern across factors, which is consistent with interpreting structural invariance in Part A as only partially supported.

For Part B, the configural model yielded fit indices at the threshold of acceptability (*χ*^2^(160) = 349.99, *p* < 0.001, CFI = 0.90, TLI = 0.87, GFI = 0.93, RMSEA = 0.10, SRMR = 0.10). Importantly, no substantial decreases in fit were observed when testing metric (ΔCFI = −0.01; ΔSRMR = 0.00) and scalar invariance (ΔCFI = 0.00; ΔSRMR = 0.00). However, the structural invariance model (*χ*^2^(215) = 478.43, *p* < 0.001, CFI = 0.86, TLI = 0.86, GFI = 0.91, RMSEA = 0.11, SRMR = 0.11) indicated a decrease in fit (ΔCFI = −0.03; ΔSRMR = +0.01), suggesting that structural invariance for Part B was only partially supported. To clarify this result, item-level diagnostics were examined for the structural model. For women, the largest modification indices were concentrated on a small subset of items, particularly DIQ_B13, DIQ_B9, DIQ_B15, and DIQ_B3, which showed the highest indices for potential cross-loadings (e.g., DIQ_B13 on the Social Misattunement and Sexual Disembodiment factors, DIQ_B9 and DIQ_B15 on the Social Misattunement factor, DIQ_B3 on the Barriers to Closeness factor). In addition, several residual covariances among items involving DIQ_B8, DIQ_B9, DIQ_B7, and DIQ_B3 (e.g., DIQ_B8–DIQ_B9, DIQ_B7–DIQ_B9, DIQ_B3–DIQ_B7, DIQ_B3–DIQ_B9) showed relatively high modification indices in the female group. For men, a similar but slightly less pronounced pattern emerged: the largest cross-loading modification indices again involved DIQ_B13 and DIQ_B9 (e.g., DIQ_B13 on the Physical Detachment and Sexual Disembodiment factors, DIQ_B9 on the Sexual Disembodiment factor), together with DIQ_B5 and DIQ_B14 on other factors, and the highest residual covariances were found for pairs such as DIQ_B8–DIQ_B9 and DIQ_B7–DIQ_B9. Overall, these local departures from invariance are limited in number and magnitude and do not form a pervasive pattern across factors, which is consistent with interpreting structural invariance in Part B as only partially supported.

The internal consistency of the DIQ proved satisfactory, with total scores showing α = 0.898 and ω = 0.913 for Part A, and α = 0.882 and ω = 0.885 for Part B (see Table 4). 

As reported in Table 4, the DIQ factors exhibited satisfactory discriminant validity, as all HTMT coefficients were below the conservative cut-off value of 0.90, indicating adequate distinctiveness among the dimensions.

Correlations between corresponding Part A and Part B subscales were medium-to-large (*r* range = 0.43–0.69), indicating substantial convergence between the mirrored perspectives, while remaining clearly below thresholds typically associated with redundancy (see Table 5).

As shown in Table 6, the DIQ total score was positively correlated with attachment anxiety (*r* = 0.447, *p* < 0.001), attachment avoidance (*r* = 0.294, *p* < 0.001), dissociation (*r* = 0.347, *p* < 0.001), alexithymia total score (*r* = 0.609, *p* < 0.001), and its subdimensions (difficulty in describing feelings, *r* = 0.549, *p* < 0.001; difficulty in identifying feelings, *r* = 0.594, *p* < 0.001; externally oriented thinking, *r* = 0.183, *p* < 0.001). Moreover, significant correlations were observed with the LPFS-BF domains of self (*r* = 0.602, *p* < 0.001) and interpersonal functioning (*r* = 0.600, *p* < 0.001). For DIQ Part A, similar associations emerged, with significant correlations with attachment anxiety (*r* = 0.461, *p* < 0.001), attachment avoidance (*r* = 0.340, *p* < 0.001), dissociation (*r* = 0.360, *p* < 0.001), alexithymia total score (*r* = 0.599, *p* < 0.001), its subdimensions (difficulty in describing feelings, *r* = 0.564, *p* < 0.001; difficulty in identifying feelings, *r* = 0.595, *p* < 0.001; externally oriented thinking, *r* = 0.139, *p* < 0.01), and the LPFS-BF self (*r* = 0.594, *p* < 0.001) and interpersonal domains (*r* = 0.560, *p* < 0.001). For DIQ Part B, significant correlations were also found with attachment anxiety (*r* = 0.344, *p* < 0.001), attachment avoidance (*r* = 0.175, *p* < 0.001), dissociation (*r* = 0.263, *p* < 0.001), alexithymia total score (*r* = 0.509, *p* < 0.001), its subdimensions (difficulty in describing feelings, *r* = 0.425, *p* < 0.001; difficulty in identifying feelings, *r* = 0.482, *p* < 0.001; externally oriented thinking, *r* = 0.210, *p* < 0.001), and the LPFS-BF self (*r* = 0.499, *p* < 0.001) and interpersonal domains (*r* = 0.543, *p* < 0.001).

## 4. Discussion

The capacity for intimacy has been highlighted as a core component of personality functioning (APA, 2013, 2022; Bender et al., 2018), and this dimension has been consistently linked in the scientific literature to strong self-perception (Price et al., 2024), relationship satisfaction (Yoo et al., 2014), and, more broadly, psychological well-being (Czyżowska et al., 2020). Given these associations, the development of reliable instruments to assess this construct is particularly important, considering its implications for both research and clinical practice. Therefore, the present study aims to develop and validate the Dissociation of Intimacy Questionnaire (DIQ), a new self-report measure designed to operationalize and assess the multifaceted construct of dissociation of intimacy.

The item generation process was carried out in the perspective of developing a mirror structure, a distinctive feature of the DIQ that captures both self- and other-representations. This bidirectional format has also been successfully employed in other multidimensional measures (e.g., Di Fabio & Gori, 2016), confirming the added value of assessing relational dynamics from complementary perspectives (“*Me with others*”/“*Others with me*”). The conceptual foundation of this approach resonates with Cooley’s (2017) notion of the “looking-glass self,” according to which the self is shaped by imagining how one is perceived by others. In this sense, the DIQ’s mirror structure provides not only a more nuanced understanding of dissociative patterns of intimacy but also clinically relevant insights into asymmetries between inner experience and interpersonal perception that may underline trauma-related disruptions in closeness (Davis et al., 2001).

In each mirroring part of the DIQ, five subscales were conceptualized, corresponding to distinct forms of dissociation of intimacy: (1) *Barriers to Closeness* reflects difficulties in expressing and sharing affective states, often rooted in fear of judgment or rejection (Giovazolias & Paschalidi, 2022); (2) *Relational Mistrust* captures impairments in reciprocal trust and empathy, consistent with attachment-based evidence showing that mistrust undermines secure emotional bonds (Gori et al., 2021; Troyer & Greitemeyer, 2018); (3) *Physical Detachment* refers to problems in tolerating bodily closeness and affectionate gestures, echoing prior studies on the embodied dimension of intimacy and its role in affective connection (Fuchs & Koch, 2014; Maclaren, 2014); (4) *Social Misattunement* involves detachment or estrangement in group contexts, aligning with theories of belongingness and relatedness as a core human motivation (Van Lange et al., 2012); (5) *Sexual Disembodiment* captures dissociative experiences specific to the sexual domain, such as derealization or external observer perspectives, which have been linked to disruptions in the integration of desire and affective presence (Bird et al., 2014; Ferreira et al., 2012). This multidimensional conceptualization was empirically supported by the psychometric findings. Both exploratory and confirmatory factor analyses confirmed the robustness of the five-factor structure, with satisfactory model fit indices. Importantly, measurement invariance analyses provided further evidence of the stability of the DIQ across genders. Configural, metric, and scalar invariance were supported, indicating that the factorial structure, the meaning of the factors, and the item intercepts are comparable in men and women (Byrne, 2012; Cheung & Rensvold, 2002; Chen, 2007). These results allow meaningful comparisons of latent means across gender, strengthening the generalizability of the instrument. However, structural invariance was not fully supported, particularly for Part B, suggesting that the strength of associations among intimacy dimensions may vary between men and women. Such findings are not uncommon in the psychometric literature (Putnick & Bornstein, 2016), and rather than undermining the validity of the instrument, they highlight the need to further explore possible gender-related differences in how intimacy processes are interconnected. Internal consistency across both the total score and the subscales was also adequate, as reflected by Cronbach’s α (Cronbach, 1951) and McDonald’s ω (McDonald, 2013) coefficients. Moreover, although the subscales were intercorrelated, their distinctiveness was confirmed through discriminant validity analyses, with HTMT coefficients below the recommended thresholds (Henseler et al., 2015). These results provide strong support for the multidimensional structure of the DIQ, demonstrating that it captures interrelated but non-redundant aspects of dissociation in intimacy.

The findings also showed significant and positive associations between the DIQ scores and the variables used to assess convergent validity. First, the DIQ scores were significantly and positively related to the insecure attachment dimensions (anxiety and avoidance). Attachment theory emphasizes how early relational experiences characterized by inconsistency, rejection, or fear undermine the capacity to experience intimacy as safe and predictable (Bowlby, 1973). In line with this, the insecure attachment patterns have been widely linked to difficulties in establishing and maintaining close and reciprocal relationships (Mikulincer & Shaver, 2016). More recent empirical findings corroborate these theoretical perspectives, showing that insecurely attached individuals are more likely to exhibit impaired intimacy, relational mistrust, and heightened relational dysregulation (Constant et al., 2021; Scigala et al., 2021; Shaver et al., 2019; Simpson & Rholes, 2017). Accordingly, the positive correlations between the DIQ and attachment anxiety and avoidance highlight that dissociation of intimacy may represent a defensive manifestation of insecure attachment strategies, reflecting a difficulty in integrating closeness with emotional safety. Consistently, significant positive correlations also emerged between the DIQ and alexithymia. Alexithymia has long been associated with impaired affect regulation and relational functioning (see Hesse & Gibbons, 2019 for a longitudinal research). The inability to recognize and communicate one’s inner states undermines emotional sharing, which is essential for the development of intimacy (Schimmenti & Caretti, 2016). Previous studies have consistently shown that alexithymia is negatively associated with interpersonal closeness and intimacy, leading to relational detachment and difficulties in co-regulation (Humphreys et al., 2009). More recent empirical work further indicates that alexithymia not only compromises emotional attunement but also increases the risk of dissociative experiences, as individuals resort to defensive strategies of disconnection when confronted with affective vulnerability (Goerlich-Dobre et al., 2015; Jordan & Smith, 2017; Nicolò et al., 2011; Schimmenti, 2017). The DIQ was also significantly and positively correlated with somatoform dissociation. Somatoform dissociation refers to disruptions in bodily perception, sensation, and motor functions that arise as defensive responses to overwhelming stress or trauma (Nijenhuis et al., 1997). This construct is particularly relevant to intimacy, since relational closeness often involves embodied presence and physical attunement. When traumatic attachment histories or unresolved affective dysregulation are present, proximity can trigger somatoform responses such as numbness, anesthesia, or detachment from the body, which hinder emotional and physical connection (Farina & Schimmenti, 2025; Schimmenti & Caretti, 2016). Prior empirical findings indicate that higher somatoform dissociation is associated with difficulties in emotional awareness, interpersonal distrust, and relational withdrawal (Şar et al., 2004; Lyssenko et al., 2018). The DIQ also showed strong correlations with the Level of Personality Functioning. According to the framework of the DSM-5 Alternative Model for Personality Disorders (APA, 2013, 2022), impairments in self and interpersonal functioning constitute the central features of personality pathology (Bender et al., 2018). Among these domains, intimacy is explicitly conceptualized as the capacity to form close and mutually satisfying relationships, whereas its disturbances manifest as detachment, mistrust, or superficiality in relational bonds. The significant associations between DIQ scores and both self- and interpersonal dysfunction suggest that dissociation of intimacy may be a fundamental marker of impaired personality functioning. Recent empirical evidence supports this view, showing that difficulties in intimacy are strongly related to maladaptive personality traits and relational dysregulation (see Di Bartolomeo et al., 2024; Jeung & Herpertz, 2014; Zimmermann et al., 2019 for reviews).

The present findings also invite a broader reflection on how dissociation of intimacy relates to contemporary models of self-experience. In line with Di Plinio et al. (2024), the sense of self can be described as emerging from the integration of identity and agency, with alterations in these components contributing to several psychopathological dimensions. Our results extend this perspective by suggesting that traumatic attachment and interpersonal mistrust may lead to a selective destabilization of self-experience within intimate relationships, rather than a generalized erosion of selfhood. From this vantage point, dissociation of intimacy can be viewed as a relational specialization of self-disturbance. The construct does not refer simply to global self-fragmentation or to broad anomalies of ipseity, but to the difficulty of sustaining a coherent sense of self-as-agent and self-as-subject in situations of emotional and physical proximity. This conceptualization helps differentiate dissociation of intimacy from related phenomena. First, compared with the global self-disturbances typically described in psychosis, dissociation of intimacy appears more context-bound and intimately linked to attachment and intimacy, which is consistent with the predominantly non-psychotic nature of our sample. Second, in contrast to measures that assess a general sense of self or anomalous self-experiences, the DIQ focuses specifically on how self-experience breaks down in intimate relationships, across emotional, psychological, physical, social, and sexual domains. Finally, the pattern of associations observed in this study—linking dissociation of intimacy with alexithymia, somatoform dissociation, and impairments in the intimacy domain of personality functioning—suggests that intimacy-related self-fragmentation may constitute one of the pathways through which developmental trauma and attachment insecurity shape adult personality organization.

### 4.1. Limitations and Suggestions for Future Research

The present study has some limitations that should be acknowledged. First, the sample was recruited through an online snowball procedure, which may restrict the generalizability of findings due to its non-random nature. Future research should consider more rigorous sampling strategies, such as random or stratified recruitment, to ensure greater representativeness across different demographic groups. Second, although measurement invariance across gender was examined and support was found for configural, metric, and scalar invariance, potential differences in the psychometric structure of the DIQ across other relevant subgroups (e.g., age groups, relationship status, sexual orientation, or clinical vs. non-clinical status) were not further explored. Given the uneven distribution of participants across these categories, subgroup sizes may limit the feasibility of adequately powered multi-group analyses. Future research is therefore encouraged to replicate and extend the present findings into more diverse and stratified samples, explicitly examining the generalizability of the scale across different demographic and clinical populations. Third, the study was conducted on a non-clinical sample, which limits the applicability of the results to clinical populations. Further research is needed to validate the DIQ in clinical groups, particularly in individuals with personality disorders, trauma histories, or dissociative conditions, where disruptions of intimacy are especially relevant. Such studies should also be used to derive clinically meaningful cut-off scores, before the DIQ can be confidently employed as a screening tool in applied settings. Fourth, all variables were assessed through self-report measures, which may be affected by social desirability, response styles such as acquiescence, and the absence of formal attention checks or social desirability scales. In addition, evidence for convergent and criterion validity was obtained from a relatively narrow set of self-report correlates (attachment, alexithymia, and personality functioning), which does not allow a full evaluation of discriminant and incremental validity. Future studies should therefore adopt broader, multi-method designs (e.g., clinician and partner ratings, behavioral indices), include explicit response-quality indicators, and test the incremental contribution of DIQ scores to predicting intimacy-related outcomes (e.g., AMPD intimacy, relationship satisfaction). Fifth, the study used a single-wave cross-sectional design, and no follow-up assessment was conducted, so test–retest reliability and temporal stability of DIQ scores could not be examined. Future research should include longitudinal designs to evaluate the stability of dissociation-of-intimacy dimensions over time. Finally, the present study examined the DIQ structure using EFA, CFA, and measurement invariance analyses, consistent with the primary goal of developing five theoretically derived and easily interpretable subscales. Other modelling approaches (e.g., ESEM, higher-order or bifactor models) were beyond the scope of this work and may be considered in future studies to further refine the structural understanding of dissociation of intimacy.

### 4.2. Clinical Relevance of the DIQ in Psychotherapy

In contemporary psychotherapy, particularly within integrative frameworks, the capacity to engage in emotionally meaningful relationships is considered a core aspect of mental health and therapeutic change (Bateman & Fonagy, 2016; Schore, 2012). Yet, many patients present with impairments in intimacy that are not readily captured by standard diagnostic tools or general symptom checklists.

The Dissociation of Intimacy Questionnaire (DIQ) offers a clinically grounded means of assessing the subtle and multifaceted ways in which intimacy may be dissociated, avoided, or disrupted, providing both a self-focused perspective (“Me with others”) and an other-focused perspective (“Others with me”).

Clinically, the five DIQ subscales map onto distinct patterns of intimacy disruption that can inform case formulation and intervention planning. Elevations on Barriers to Closeness suggest difficulties in sharing inner experiences and tolerating affective closeness, pointing to the usefulness of interventions that foster emotional awareness, mentalization, and affect regulation within the therapeutic relationship (e.g., Bateman & Fonagy, 2016). High scores on Relational Mistrust indicate pervasive expectations of unreliability, rejection, or betrayal, which may call for a strong focus on pacing, alliance building, and the exploration of core beliefs about others and the self in relationships. Elevations on Physical Detachment highlight patterns of bodily withdrawal or reduced comfort with physical proximity, which can be addressed through work on embodied presence, grounding, and the regulation of autonomic arousal (Schore, 2012; Ogden et al., 2006). High scores on Social Misattunement point to difficulties in reading, anticipating, or coordinating with others in social contexts, suggesting the relevance of interventions targeting social cognition, perspective-taking, and interpersonal skills. Finally, marked elevations on Sexual Disembodiment may signal dissociated, avoidant, or conflictual experiences of sexuality and the body, and can cue more targeted psychosexual, trauma-informed, or body-oriented interventions, including careful exploration of sexual history, consent, and safety.

The mirrored structure of the DIQ further allows clinicians to compare “Me with others” and “Others with me” scores within each domain, highlighting potential asymmetries between self-perceptions and perceptions of others’ intentions or behaviors. For example, a discrepancy in which a patient reports high Relational Mistrust in Part A but perceives relatively benign behavior from others in Part B may suggest the predominance of internalized expectations of danger, whereas the opposite pattern may indicate exposure to genuinely unreliable or invalidating contexts. Such information can help therapists differentiate between primarily intrapsychic schemas and current relational realities and to anticipate how these patterns may be enacted in transference and countertransference (Bromberg, 1998, 2006; Loewenstein, 2018). In addition, the DIQ can support alliance and attunement by offering therapists a structured map of the client’s subjective experience of closeness and distance across emotional, physical, social, and sexual domains. This can help identify areas where proximity may be experienced as neuroceptively dangerous and where autonomic defense responses (e.g., fight, flight, or freeze) are likely to be activated (Griffies, 2019; Porges, 2011, 2021; Ogden et al., 2006). Finally, although normative cut-offs are not yet available, repeated administrations within the same individual can be used to monitor therapeutic change over time. Tracking reductions in dissociative responding and increases in relational openness across the DIQ subscales may help clinicians evaluate progress not only in terms of symptom reduction but also in terms of improved capacity for intimacy and co-regulation (Brewer & Giommi, 2025; Newhill et al., 2003; Wilcox & Almasifard, 2023).

## 5. Conclusions

The present study introduced and validated the Dissociation of Intimacy Questionnaire (DIQ; see Appendix A and Appendix B), a multidimensional instrument for assessing the dissociative and defensive processes that compromise intimacy. Through its two parallel forms (“Me with others” and “Others with me”) and five distinct dimensions (emotional, psychological, physical, social, and sexual), the DIQ captures the complexity of how intimacy can be disrupted or fragmented. This structure provides a clinically rich framework for identifying patterns of disconnection in both clinical and non-clinical populations. Beyond its psychometric soundness, the DIQ holds significant clinical value. By illuminating subtle disruptions in closeness, the instrument can guide case formulation, inform therapeutic attunement, and monitor progress over time. In this sense, the DIQ is not only a diagnostic tool but also a clinical compass, orienting therapists toward the embodied, affective, and relational dimensions of dissociation.

## Figures and Tables

**Table 1:** Socio-demographic characteristics of the sample (*N* = 500).

Variable	*M* ± *SD*	Categories	*n*	%
Age	31.92 ± 12.78			
Sex		Women	370	74
		Men	130	26
Marital status		Single	345	69
		Cohabiting	62	12.4
		Married	70	14
		Divorced	14	2.8
		Separated	8	1.6
		Widowed	1	0.2
Educational level		Middle school	12	2.4
		High school diploma	174	34.8
		Bachelor’s degree	118	23.6
		Master’s degree	126	25.2
		Post-graduate specialization	70	14
Employment status		Student	164	32.8
		Working student	99	19.8
		Employee	115	23
		Freelancer	79	15.8
		Entrepreneur	11	2.2
		Manager	7	1.4
		Unemployed	14	2.8
		Retired	9	1.8
		Artisan	1	0.2
		Trader	1	0.2

**Table 2:** Factor structure matrix for the two parts of the DIQ.

Items	DIQ Part A (Me with Others)					Items	DIQ Part B (Others with Me)				
	Barriers to Closeness	Relational Mistrust	Physical Detachment	Social Misattunement	Sexual Disembodiment		Barriers to Closeness	Relational Mistrust	Physical Detachment	Social Misattunement	Sexual Disembodiment
DIQ_A1	0.699					DIQ_B1	0.521				
DIQ_A2	0.315					DIQ_B2	0.459				
DIQ_A3	0.616					DIQ_B3	0.808				
DIQ_A4		0.665				DIQ_B4		0.783			
DIQ_A5		0.845				DIQ_B5		0.677			
DIQ_A6		0.529				DIQ_B6		0.318			
DIQ_A7			0.705			DIQ_B7			0.673		
DIQ_A8			0.878			DIQ_B8			0.771		
DIQ_A9			0.612			DIQ_B9			0.750		
DIQ_A10				0.624		DIQ_B10				0.502	
DIQ_A11				0.727		DIQ_B11				0.431	
DIQ_A12				0.752		DIQ_B12				0.427	
DIQ_A13					0.810	DIQ_B13					0.831
DIQ_A14					0.646	DIQ_B14					0.733
DIQ_A15					0.723	DIQ_B15					0.684

**Table 3:** Fit indices and multigroup invariance analyses of the DIQ.

	*χ* ^2^	*df*	*p*	CFI	TLI	GFI	RMSEA	SRMR	ΔCFI	ΔSRMR
***Confirmatory factor analysis***										
Part A	131.017	80	0.001	0.98	0.97	0.99	0.05	0.06		
Part B	173.610	80	0.001	0.95	0.93	0.99	0.07	0.06		
***Multigroup analyses to test the measurement invariance in Part A***										
Configural invariance	311.740	160	0.001	0.93	0.90	0.94	0.09	0.06	-	-
Metric invariance	333.867	170	0.001	0.92	0.90	0.93	0.09	0.08	−0.02	−0.02
Scalar invariance	343.859	180	0.001	0.92	0.91	0.93	0.09	0.07	0.00	−0.01
Structural invariance	399.917	195	0.001	0.91	0.91	0.92	0.08	0.11	−0.01	0.04
***Multigroup analyses to test the measurement invariance in Part B***										
Configural invariance	349.989	160	0.001	0.90	0.87	0.93	0.10	0.10	-	-
Metric invariance	366.864	170	0.001	0.89	0.87	0.93	0.10	0.10	−0.01	0.00
Scalar invariance	391.650	180	0.001	0.89	0.87	0.93	0.10	0.10	0.00	0.00
Structural invariance	478.432	215	0.001	0.86	0.86	0.91	0.10	0.11	−0.03	0.01

***Note***. *χ^2^ = chi-square value of model fit; df = degrees of freedom; CFI = Comparative Fit Index; TLI = Tucker–Lewis Index; GFI = Goodness-of-Fit Index; RMSEA = Root Mean Square Error of Approximation; SRMR = Standardized Root Mean Square Residual; ΔCFI = difference in CFI between the compared models; ΔSRMR = difference in SRMR between the compared models.*

**Table 4:** Internal consistency, Inter-Factor Correlations, and HTMT analysis for the DIQ.

Part	Dimensions	Items (N)	α	ω	Inter-Factor Correlations (Above the Diagonal) and HTMT Analysis (Below the Diagonal).				
					**1**	**2**	**3**	**4**	**5**
A	Total	15	0.898	0.913					
	1. Barriers to Closeness	3	0.758	0.775	1	0.673	0.762	0.642	0.623
	2. Relational Mistrust	3	0.761	0.771	0.612	1	0.472	0.659	0.420
	3. Physical Detachment	3	0.869	0.872	0.748	0.452	1	0.618	0.942
	4. Social Misattunement	3	0.802	0.802	0.521	0.569	0.533	1	0.583
	5. Sexual Disembodiment	3	0.846	0.849	0.573	0.396	0.879	0.507	1
B	Total	15	0.882	0.885					
	1. Barriers to Closeness	3	0.722	0.735	1	0.710	0.625	0.728	0.526
	2. Relational Mistrust	3	0.665	0.674	0.583	1	0.634	0.816	0.536
	3. Physical Detachment	3	0.813	0.818	0.615	0.578	1	0.823	0.895
	4. Social Misattunement	3	0.703	0.692	0.621	0.747	0.714	1	0.822
	5. Sexual Disembodiment	3	0.840	0.843	0.472	0.482	0.803	0.695	1

***Note****:* All Pearson inter-factor correlations were significant at the *p* < 0.01 level. DIQ Part A = Dissociation of Intimacy (Me with others); DIQ Part B = Dissociation of Intimacy (Others with me).

**Table 5:** Pearson’s Correlations between the total and the factors of Part A (horizontal axis) and Part B (vertical axis).

Variable	DIQ Part A	Barriers to Closeness	Relational Mistrust	Physical Detachment	Social Misattunement	Sexual Disembodiment
DIQ Part B	0.691 ***	0.569 ***	0.527 ***	0.582 ***	0.632 ***	0.582 ***
Barriers to Closeness	0.532 ***	0.427 ***	0.481 ***	0.401 ***	0.499 ***	0.408 ***
Relational Mistrust	0.555 ***	0.457 ***	0.480 ***	0.417 ***	0.576 ***	0.417 ***
Physical Detachment	0.579 ***	0.477 ***	0.409 ***	0.513 ***	0.515 ***	0.519 ***
Social Misattunement	0.693 ***	0.555 ***	0.528 ***	0.584 ***	0.668 ***	0.585 ***
Sexual Disembodiment	0.567 ***	0.466 ***	0.336 ***	0.538 ***	0.496 ***	0.545 ***

***Note***. Bold values indicate correlations between mirroring scales. *** *p* < 0.001. DIQ Total Score = Dissociation of Intimacy; DIQ Part A = Dissociation of Intimacy (Me with others); DIQ Part B = Dissociation of Intimacy (Others with me).

**Table 6:** Correlation Matrix.

Variable	Insecure Attachment (RQ)		Dissociation (SDQ—5)	Alexithymia (TAS-20)				Level of Personality Functioning Scale (LPFS-BF)	
	Attachment Anxiety	Attachment Avoidance		Tot	Difficulty in Describing Feelings	Difficulty in Identifying Feelings	Externally Oriented Thinking	Self	Interpersonal
DIQ Total score	0.447 ***	0.294 ***	0.347 ***	0.609 ***	0.549 ***	0.594 ***	0.183 ***	0.602 ***	0.600 ***
DIQ Part A	0.461 ***	0.340 ***	0.360 ***	0.599 ***	0.564 ***	0.595 ***	0.139 **	0.594 ***	0.560 ***
Barriers to Closeness	0.393 ***	0.392 ***	0.242 ***	0.636 ***	0.678 ***	0.528 ***	0.212 ***	0.524 ***	0.467 ***
Relational Mistrust	0.498 ***	0.315 ***	0.302 ***	0.496 ***	0.491 ***	0.486 ***	0.099 *	0.523 ***	0.516 ***
Physical Detachment	0.325 ***	0.271 ***	0.292 ***	0.481 ***	0.434 ***	0.483 ***	0.124 **	0.479 ***	0.437 ***
Social Misattunement	0.354 ***	0.228 ***	0.333 ***	0.475 ***	0.420 ***	0.502 ***	0.093 *	0.493 ***	0.496 ***
Sexual Disembodiment	0.299 ***	0.212 ***	0.324 ***	0.430 ***	0.355 ***	0.476 ***	0.082	0.460 ***	0.422 ***
DIQ Part B	0.344 ***	0.175 ***	0.263 ***	0.509 ***	0.425 ***	0.482 ***	0.210 ***	0.499 ***	0.543 ***
Barriers to Closeness	0.339 ***	0.127 **	0.203 ***	0.417 ***	0.328 ***	0.401 ***	0.185 ***	0.415 ***	0.511 ***
Relational Mistrust	0.294 ***	0.121 **	0.211 ***	0.434 ***	0.389 ***	0.389 ***	0.182 ***	0.449 ***	0.479 ***
Physical Detachment	0.228 ***	0.171 ***	0.175 ***	0.422 ***	0.352 ***	0.356 ***	0.236 ***	0.377 ***	0.412 ***
Social Misattunement	0.347 ***	0.156 ***	0.300 ***	0.502 ***	0.421 ***	0.492 ***	0.180 ***	0.513 ***	0.546 ***
Sexual Disembodiment	0.197 ***	0.165 ***	0.193 ***	0.407 ***	0.322 ***	0.382 ***	0.192 ***	0.362 ***	0.360 ***

***Note***. * *p* < 0.05, ** *p* < 0.01, *** *p* < 0.001. DIQ Total Score = Dissociation of Intimacy; DIQ Part A = Dissociation of Intimacy (Me with others); DIQ Part B = Dissociation of Intimacy (Others with me).

## Data Availability

Data presented in this study are available on request from the corresponding author.

## Appendix A. Dissociation of Intimacy Questionnaire (DIQ)—Italian

Di seguito sono messe in evidenza alcune modalità che riguardano il modo di relazionarsi e di essere con le persone. Nella fattispecie queste affermazioni si riferiscono a come le persone percepiscono loro stesse e gli altri nelle relazioni strette o d’intimità. Nella prima parte può descrivere come lei si pone abitualmente con gli altri. Nella seconda parte può descrivere come gli altri si pongono abitualmente o si sono posti con lei (può considerare come riferimento temporale le ultime quattro settimane). Risponda con una crocetta a tutte le affermazioni esprimendo la sua preferenza scegliendo tra: **(1)** Per Niente **(2)** Poco **(3)** Abbastanza **(4)** Molto **(5)** Moltissimo. La preghiamo di completare per intero entrambe le parti.

**(a) Io Con Gli Altri**							**(b) Gli Altri Con Me**						
1a	Nelle situazioni di intimità trovo difficoltà a parlare dei miei sentimenti	1	2	3	4	5	1b	Nelle situazioni di intimità ho l’impressione che gli altri abbiano difficoltà a parlare dei loro sentimenti con me	1	2	3	4	5
2a	Evito di coinvolgermi in relazioni sentimentali	1	2	3	4	5	2b	Gli altri evitano di coinvolgersi in relazioni sentimentali con me	1	2	3	4	5
3a	Mi viene difficile condividere con gli altri ciò che provo	1	2	3	4	5	3b	Gli altri hanno difficoltà a condividere ciò che provano con me	1	2	3	4	5
4a	Mi viene difficile fidarmi degli altri	1	2	3	4	5	4b	Gli altri hanno difficoltà a fidarsi di me	1	2	3	4	5
5a	Sono sempre sospettoso, anche con persone che conosco da tempo	1	2	3	4	5	5b	Gli altri sono sempre sospettosi con me, anche coloro che mi conoscono da tempo	1	2	3	4	5
6a	Quando sono innamorato temo di essere abbandonato	1	2	3	4	5	6b	Quando qualcuno è innamorato di me, teme di essere abbandonato	1	2	3	4	5
7a	In situazioni di intimità, ho difficoltà ad entrare in contatto fisico con l’altro	1	2	3	4	5	7b	In situazioni d’intimità, gli altri hanno difficoltà ad entrare in contatto fisico con me	1	2	3	4	5
8a	In situazioni di intimità fisica tendo a non essere del tutto presente	1	2	3	4	5	8b	In situazioni di intimità fisica con me, percepisco gli altri come non del tutto presenti	1	2	3	4	5
9a	Nei contesti che implicano vicinanza fisica, mi sento come se fossi distaccato	1	2	3	4	5	9b	Nei contesti che implicano vicinanza fisica, sento gli altri come se fossero distaccati	1	2	3	4	5
10a	In generale quando gli altri si divertono io mi annoio	1	2	3	4	5	10b	In generale ho l’impressione che gli altri si annoino quando io mi diverto	1	2	3	4	5
11a	Quando sono con gli altri mi sembra di essere uno spettatore estraneo di quello che succede	1	2	3	4	5	11b	Quando gli altri sono con me, li percepisco come se fossero degli spettatori estranei rispetto a ciò che succede	1	2	3	4	5
12a	Quando sono con gli altri non vedo l’ora di andarmene	1	2	3	4	5	12b	Quando gli altri sono con me è come se non vedessero l’ora di andarsene	1	2	3	4	5
13a	Nell’intimità sessuale mi capita di non sentire le mie sensazioni corporee	1	2	3	4	5	13b	Nelle situazioni di intimità sessuale con me percepisco gli altri come se non riuscissero a sentire le loro sensazioni corporee	1	2	3	4	5
14a	Nei momenti di intimità sessuale mi sembra che quell’esperienza non sia completamente reale	1	2	3	4	5	14b	Nei momenti d’intimità sessuale con me, percepisco gli altri come se vivessero quell’esperienza in modo non completamente reale	1	2	3	4	5
15a	Nell’intimità sessuale, talvolta mi sento come se fossi uno spettatore esterno	1	2	3	4	5	15b	Nelle situazioni di intimità sessuale con me, mi capita di percepire gli altri come se fossero degli spettatori esterni	1	2	3	4	5

## Appendix B. Dissociation of Intimacy Questionnaire (DIQ)—English Translation

Below are highlighted some ways of relating and being with people. Specifically, these statements refer to how individuals perceive themselves and others in their close or intimate relationships. In the first part, you may describe how you usually behave with others. In the second part, you may describe how others usually behave, or have behaved, with you (you may consider the last four weeks as a reference period). Please respond to all statements by ticking your preference, choosing among: **(1)** Not at all **(2)** A little **(3)** Moderately **(4)** Quite a lot **(5)** Very much. We kindly ask you to fully complete both parts.

**(a) Myself with Others**							**(b) Others with Me**						
1a	In situations of intimacy, I find it difficult to talk about my feelings.	1	2	3	4	5	1b	In situations of intimacy, I have the impression that others find it difficult to talk about their feelings with me.	1	2	3	4	5
2a	I avoid getting involved in romantic relationships.	1	2	3	4	5	2b	Others avoid getting involved in romantic relationships with me.	1	2	3	4	5
3a	I find it difficult to share with others what I feel.	1	2	3	4	5	3b	Others find it difficult to share what they feel with me.	1	2	3	4	5
4a	I find it difficult to trust others.	1	2	3	4	5	4b	Others find it difficult to trust me.	1	2	3	4	5
5a	I am always suspicious, even with people I have known for a long time.	1	2	3	4	5	5b	Others are always suspicious of me, even those who have known me for a long time.	1	2	3	4	5
6a	When I am in love, I am afraid of being abandoned.	1	2	3	4	5	6b	When someone is in love with me, they are afraid of being abandoned.	1	2	3	4	5
7a	In situations of intimacy, I find it difficult to make physical contact with the other person.	1	2	3	4	5	7b	In situations of intimacy, others find it difficult to make physical contact with me.	1	2	3	4	5
8a	In situations of physical intimacy, I tend not to be fully present.	1	2	3	4	5	8b	In situations of physical intimacy with me, I perceive others as not fully present.	1	2	3	4	5
9a	In contexts where physical proximity is involved, I feel as if I were detached.	1	2	3	4	5	9b	In contexts where physical proximity is involved, I feel as if others were detached.	1	2	3	4	5
10a	In general, I feel bored when others are having fun.	1	2	3	4	5	10b	In general, I have the impression that others feel bored when I am having fun.	1	2	3	4	5
11a	When I am with others, I feel like an external spectator of what is happening.	1	2	3	4	5	11b	When others are with me, I feel like they are external spectators of what is happening.	1	2	3	4	5
12a	When I am with others, I can’t wait to leave.	1	2	3	4	5	12b	When others are with me, I feel like they can’t wait to leave.	1	2	3	4	5
13a	In sexual intimacy, I sometimes do not feel my bodily sensations.	1	2	3	4	5	13b	In situations of sexual intimacy with me, I perceive others as if they were unable to feel their bodily sensations.	1	2	3	4	5
14a	In moments of sexual intimacy, I feel like that experience is not entirely real.	1	2	3	4	5	14b	In moments of sexual intimacy with me, I perceive others as if they are experiencing it as not entirely real.	1	2	3	4	5
15a	In sexual intimacy, at times I feel as if I were an external spectator.	1	2	3	4	5	15b	In situations of sexual intimacy with me, I sometimes perceive others as if they were external spectators.	1	2	3	4	5
